# Analyzing Tweeting Patterns and Public Engagement on Twitter During the Recognition Period of the COVID-19 Pandemic: A Study of Two U.S. States

**DOI:** 10.1109/access.2022.3189670

**Published:** 2022

**Authors:** MISBAH UL HOQUE, KISUNG LEE, JESSICA L. BEYER, SARA R. CURRAN, KATIE S. GONSER, NINA S. N. LAM, VOLODYMYR V. MIHUNOV, KEJIN WANG

**Affiliations:** 1 Division of Computer Science and Engineering, Louisiana State University, Baton Rouge, LA 70803, USA; 2 The Henry M. Jackson School of International Studies, University of Washington, Seattle, WA 98195, USA; 3 Department of Environmental Sciences, Louisiana State University, Baton Rouge, LA 70803, USA

**Keywords:** COVID-19, geospatial data analysis, natural language processing, public engagement, public health messaging, sentiment analysis, statistical analysis, Twitter data analytics

## Abstract

The abundance of available information on social media can provide invaluable insights into people’s responses to health information and public health guidance concerning COVID-19. This study examines tweeting patterns and public engagement on Twitter, as forms of social media, related to public health messaging in two U.S. states (Washington and Louisiana) during the early stage of the pandemic. We analyze more than 7M tweets and 571K COVID-19-related tweets posted by users in the two states over the first 25 days of the pandemic in the U.S. (Feb. 23, 2020, to Mar. 18, 2020). We also qualitatively code and examine 460 tweets posted by selected governmental official accounts during the same period for public engagement analysis. We use various methods for analyzing the data, including statistical analysis, sentiment analysis, and word usage metrics, to find inter- and intra-state disparities of tweeting patterns and public engagement with health messaging. Our findings reveal that users in Washington were more active on Twitter than users in Louisiana in terms of the total number and density of COVID-19-related tweets during the early stage of the pandemic. Our correlation analysis results for counties or parishes show that the Twitter activities (tweet density, COVID-19 tweet density, and user density) were positively correlated with population density in both states at the 0.01 level of significance. Our sentiment analysis results demonstrate that the average daily sentiment scores of all and COVID-19-related tweets in Washington were consistently higher than those in Louisiana during this period. While the daily average sentiment scores of COVID-19-related tweets were in the negative range, the scores of all tweets were in the positive range in both states. Lastly, our analysis of governmental Twitter accounts found that these accounts’ messages were most commonly meant to spread information about the pandemic, but that users were most likely to engage with tweets that requested readers take action, such as hand washing.

## I. INTRODUCTION

During the ongoing COVID-19 pandemic, many people have turned to online social media platforms such as Twitter to receive the latest updates regarding the pandemic and share their thoughts and responses with their followers or friends. For example, when the World Health Organization (WHO) declared COVID-19 a global pandemic on March 11, 2020, Twitter recorded an increase in users and activity.^1^ Governmental officials (including federal, state, and municipal leaders) and public health experts have used online social media to announce public policies, such as stay-at-home orders and mask wearing, and provide information about how to help prevent the spread of COVID-19. Traditional media outlets including national newspaper publishers, cable and network news, and local TV stations have also been posting news articles about the pandemic on various social media platforms.

The plethora of information available via online social media can provide invaluable insights into users’ responses to health information and public health guidance concerning COVID-19. Unlike traditional research methods, such as online surveys and phone interviews, that can be conducted over a certain period after an event to understand public opinion about the event, online social media data can include real-time responses of users when an event occurs or a new policy is announced. Researchers have analyzed social media data, including extracting sentiment patterns from social media [1]–[3] and analyzing Twitter user characteristics about using controversial COVID-19 terms [4], to understand the opinions posted on social media platforms during the pandemic.

In this study, we analyze Twitter data posted during the early stage of the COVID-19 pandemic in two U.S. states, Washington (WA) and Louisiana (LA). The early stage represents a critical time frame for public health because the public’s initial understandings of, and reactions to, the pandemic can influence subsequent behaviors during the later stages of the pandemic [5]. We define the early stage as the 25 days (Feb. 23, 2020 - Mar. 18, 2020), spanning the time in the U.S. just prior to the start of the first wave of the pandemic and just before the peak of the first wave, and call it the recognition period. This specific period was chosen in this study to understand Twitter users’ sentiment and public health messaging activity on Twitter before the public witnessed the imminent threat of the pandemic in their neighborhood.

We select the two states because they are geographically and politically different. Louisiana is located along the Gulf of Mexico, and there is reasonable suspicion that 2020’s Mardi Gras was a super-spreader event of COVID-19. Washington State is located in the Pacific Northwest region, and the state reported the first known COVID-19 case in the U.S. Although LA is considered politically conservative, having voted for Republican candidates in the last six presidential elections and WA is considered politically liberal, having voted for Democrat candidates in the last nine presidential elections, both states divide between left-voting urban and right-voting rural communities.

The main goal of this study is to examine tweeting patterns and engagement related to public health messaging in the two states during the recognition period. Because tweeting patterns and public health messaging can differ for various geographical granularities such as states and counties, this study is primarily motivated to analyze and compare tweeting patterns in the two states to understand both inter- and intra-state disparities in the use of Twitter during the pandemic. The identified geographical disparities can be used when designing more localized, effective public health messaging on social media. The results can demonstrate the value of studying social perceptions, such as sentiment changes in response to the pandemic at a finer geographical resolution.

In addition, it is important to examine how state officials or agencies communicate with their stakeholders on Twitter because sharing timely and accurate information from trustworthy sources is critical during disasters. During the early stage of the COVID-19 pandemic, timely communication from dependable sources was particularly essential because of widespread misinformation and disinformation on social media. The analysis results can provide vital insights for future governmental communication strategies such as message functions, to improve public engagement on Twitter for broader acceptance of public health messages. We pose the following research questions:

1. RQ1: How did Twitter activity, sentiment, and word usage vary in the two states?
2. RQ2: To what extent did Twitter activity and sentiment vary across counties or parishes in each state?
3. RQ3: How did tweeting patterns differ for COVID-19-related tweets in the two states?
4. RQ4: To what extent did governmental Twitter accounts communicate public health messages and did the users on Twitter engage with the messages?
5. RQ5: How did sentiment of responses to governmental COVID-19-related messages vary across both states?

Based on the research questions, we pose the following hypotheses to guide our analyses:

1. H1: Given that Washington State had the first known case of COVID-19 in the U.S., at the state level, we expect Washington State to have higher COVID-19-related tweet intensity.
2. H2: However, at the county/parish level, we expect to find the same pattern of Twitter use disparities between urban and rural found in previous studies [6] in both states.
3. H3: We expect that in counties or parishes with higher socioeconomic conditions, users will be more optimistic about the pandemic.
4. H4: Among the three message functions (action, information, and community) of the government accounts, we expect that action tweets will receive the most engagement as reported in previous studies [7], [8].

Although existing research provides some interesting observations in their analysis of social media data related to COVID-19, there are several limitations based on their data preparation and analysis. First, most existing research collected social media data using certain keywords related to the pandemic such as “covid” and “coronavirus” [3], [9]–[11]. Such filtered data can only provide insights about COVID-19-related tweets. Data prepared by considering only certain keywords can hardly express overall opinions and attitudes during the pandemic because tweets that do not explicitly mention such terms are excluded. Second, most existing works use social media data collected with a relatively small sampling ratio such as 1% sampled Twitter data. Such highly selected data may provide insights for coarse-grained analysis like country- and state-level comparisons, but it is hard to conduct finer-grained comparisons at finer levels such as county- and city-level analysis because of a lack of sufficient data. Last but not least, to the best of our knowledge, there is no existing work that analyzes social media posts directly responding to public health messages from government officials. Although some existing works analyze engagement numbers, such as retweet counts, of tweets posted by government officials during the pandemic [7], [8], they do not examine the content of engagement tweets posted by the users such as replies. It is imperative to analyze such targeted social media data to better understand public sentiment related to public health messaging during the pandemic.

This paper makes the following contributions. First, we analyze and compare tweeting patterns including sentiment and word usage in the two states to understand interstate disparities. Unlike existing works that analyze tweets selected using certain keywords, we avoid selection bias because we examine all user-posted tweets in both states without filtering using keywords. We also compare tweeting patterns between COVID-19-related tweets and all background tweets. Second, we analyze and visualize tweeting patterns and sentiment variations across counties or parishes to identify intra-state disparities. The results provide a more detailed comparison based on Twitter users’ geographical location in each state. Third, we examine the public health messaging of governmental accounts in the two states and public engagement related to the messages. Specifically, we analyze the tweeting patterns of selected accounts and their public engagement metrics based on message functions to understand the characteristics of messages that generated higher interaction. We also measure the sentiment of direct replies to governmental public health tweets related to the pandemic.

The rest of the paper is organized as follows. We summarize the related works of this study in Sec. II. Sec. III introduces our overall data processing pipeline used in this study. Sec. IV describes tweeting patterns in the two states during the recognition period. Sec. V presents sentiment analysis results for both inter- and intra-state comparisons. In Sec. VI, we describe the tweeting patterns of selected governmental accounts and users’ engagement with their public health messaging. We discuss our research findings based on proposed research questions and hypotheses in Sec. VII and conclude the paper in Section VIII.

## II. RELATED WORKS

### A. SENTIMENT ANALYSIS

Communication has largely shifted to online social media during the virus containment period of the pandemic. Growing social media use during the pandemic necessitates the analysis of social media data to extract social behavior patterns of the public so that officials can design policies accordingly. Recent research shows that sentiment analysis is a salient approach to measuring social responses on online social media. Alzamzami and Saddik [12] propose an unsupervised and supervised approach for data discovery and social behavior analysis for 12 months in Canada and U.S. during the pandemic. They use two supervised classifiers based on Bidirectional Encoder Representations from Transformers (BERT) to analyze two types of social behavior, hate and sentiment. A binary classifier is used to detect hate, and a multi-class classifier is used to detect sentiment. For data exploration and coherent interpretation, the study uses an unsupervised framework. Imran *et al.* [13] analyze public reaction and sentiment related to different actions during the pandemic at country-level granularity. They use a trained deep long short-term memory (LSTM) model on extracted tweets for the estimation of sentiment polarity and emotions. The study finds a high correlation between sentiment expressed in neighboring countries (e.g., U.S. and Canada). Vyas *et al.* [14] conduct a comparative performance study among supervised machine learning (ML) techniques for sentiment analysis on COVID-19-related tweets. They use a hybrid framework combining lexicon-based Valence Aware Dictionary and sEntiment Reasoner (VADER) and supervised ML (e.g., Naive Bayes, Decision Tree, Random Forest, Logistic Regression, and LSTM). They run VADER to label the sentiment polarity of tweets and use the labels for training ML approaches. Topbas *et al.* [15] use two deep learning models, Recurrent Neural Network (RNN) and BERT, for sentiment analysis of COVID-19-related tweets. Their experimental results show that the effective accuracy of the models is above 83%.

Our study differs from the above research works in terms of data collection, data preprocessing, and sentiment analysis methods. Most of the recent research works such as [13], [14], and [15] collected Twitter data that contains specific keywords like “coronavirus” and “COVID-19”. We have not used any keywords for collecting data to avoid selection bias. [12], [13], and [15] used data preprocessing such as converting uppercase to lowercase and removing emojis, emoticons, intensifier words, and acronyms. Such data preprocessing steps on Twitter data can cause a significant loss of sentiment-related information from the tweet text. For example, emojis, intensifier words like “extremely”, and capitalized words like “GREAT” can express different sentiment levels. We avoid such data preprocessing and use VADER for sentiment analysis, which can perform well on emojis, emoticons, capitalization, intensifiers, acronyms, etc.

### B. PUBLIC ENGAGEMENT ANALYSIS

Public engagement analysis helps decision-makers discover emerging concerns and opinions that could mitigate the bidirectional communication gap between the public and officials during crises such as natural disasters and pandemics. In [7], the authors use a mixed-method approach to examine the content and engagement of COVID-19-related tweets posted by Canadian health agencies and officials. The study reveals that the largest percentage of COVID-19-related tweets were authored by public health officials, and they received the highest public engagement in terms of retweets per tweet. The study also finds that, while the information function is the most commonly used message function, tweets promoting the action function received the highest public interaction in terms of retweets and likes. In a related work [8], the authors use natural language processing (NLP) and hierarchical linear regression to examine the content and the level of public engagement of COVID-19-related tweets posted by public agencies in Texas, U.S. Similar to [7], findings of the study show that the information function was dominant among other message functions during the study period. The study suggests that tweets were more likely to be retweeted when they were related to the information and action functions while tweets were more likely to be liked when they were related to the action and community functions. Alhassan and AlDossary [16] conduct a related study to explore the Twitter use and public engagement of the Ministry of Health in Saudi Arabia during the pandemic. They analyze public engagement in terms of retweets and likes, similar to [7] and [8]. Their finding suggests that warning-, uncertainty reduction-, and reassurance-related tweets gained the highest levels of public engagement. The study also shows that the inclusion of hyperlinks and media files negatively correlates with public engagement, and the use of hashtags is positively correlated with public engagement.

## III. TWITTER DATA COLLECTION AND PROCESSING

### A. DATA COLLECTION AND PROCESSING FRAMEWORK

**FIGURE 1.**
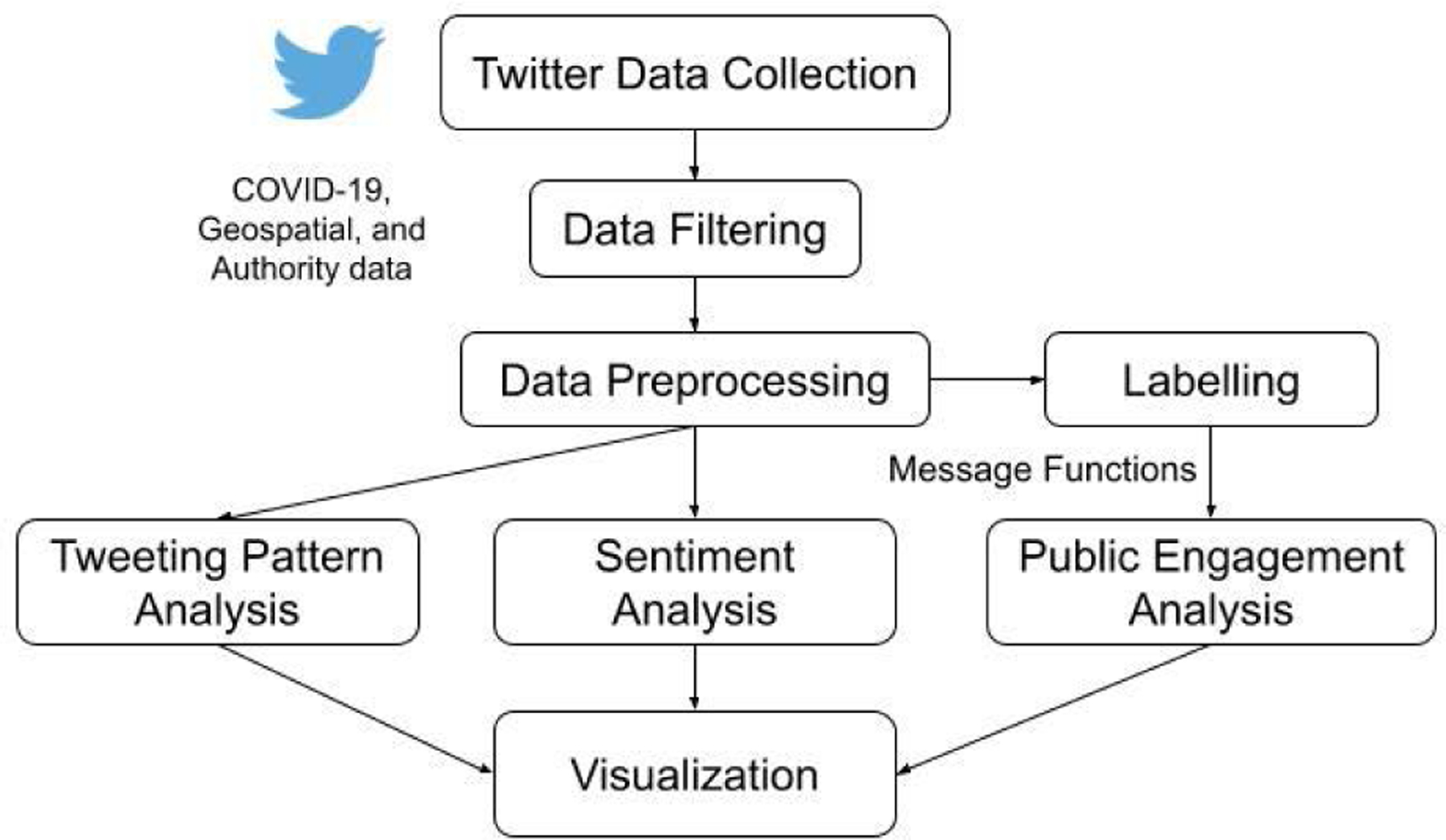
Twitter Data Collection and Processing Framework.

Fig. 1 presents our framework to collect and process Twitter data for this study. First, we collect tweets posted by Twitter accounts located in WA and LA. To collect and extract the Twitter data, we utilize two types of Twitter APIs, Historical PowerTrack and Academic Research Track.^2^ We store collected Twitter data using MongoDB, a NoSQL data store optimized for document-style data such as JSON. Second, we need to filter useful data from collected raw data for specific analysis tasks. We use several ETL (Extract-Transform-Load) programs to extract necessary data from the MongoDB collections and transform the data into appropriate formats for downstream analytical tasks and visualization. We use language and geospatial operators to filter all English tweets and users located in either WA or LA. To find COVID-19-related tweets, we use a regular expression based on 29 COVID-19-related keywords. To analyze tweets from governmental accounts, we also manually label the message function of each tweet. Third, we use data preprocessing steps for each filtered data separately according to task-specific requirements. Then, we perform tweet pattern, sentiment, and public engagement analysis on the filtered and preprocessed data. Finally, we visualize results from each analysis and present research findings to answer proposed research questions. We describe the details of the framework in the following sections.

### B. DATA COLLECTION

We define the recognition period of the pandemic as 25 days from Feb. 23, 2020, to Mar. 18, 2020, in this study. As shown in Table 1, this period covers more than three weeks before the governor in each state issued a “stay at home” order. This period also includes the date when the first COVID-19 death was reported in each state. By focusing on this period, we aim to understand the tweeting patterns of users in the two states and their engagement related to public health messaging before the public experienced tangible restrictions such as stay-at-home requirements.

To collect tweets posted by users located in the two states during the recognition period, we use geospatial operators^3^ through the Historical PowerTrack API of Twitter. The primary operator we use for this collection is profile_region:, which finds tweets with the specified region (corresponding to states in the U.S.) in the profile location. Unlike other spatial operators that are directly matched against the user-generated and noisy location field such as bio_location:, the operator utilizes structured geo-data derived by Twitter, called Profile Geo Enrichment.^4^ We also specify additional rules using a tweet-level location operator (place:) and user profile-level operators (bio_location: and bio:) using state names and abbreviations to capture more tweets. We also use 50% as our sampling ratio for this data collection, and so we assume that most active users located in LA or WA based on the derived location could be captured during our 25-day study period. For example, if a user posted ten tweets (including retweets, replies, and quoted tweets) during the period, the probability that at least one tweet of the user is included in our collection is higher than 99.9% (i.e., 1 − 0.5^10^). In this paper, we report analysis results of tweets written in English and posted by users located in Louisiana or Washington based on the derived user location. In addition, to extract complete tweets from specific Twitter handles and tweet-level metrics, we use the Academic Research Track API, which supports the full-archive search API.

### C. SENTIMENT SCORES

Sentiment analysis is a popular text classification technique that analyzes the underlying sentiment of a text (e.g., positive, negative, neutral). In this study, we conduct sentiment analysis to gain an understanding of overall opinions and attitudes on Twitter. VADER [17] has been widely used to extract the sentiment scores of social media posts, and a recent study even reports that it works better than some state-of-the-art deep learning-based prediction models for analyzing tweets related to COVID-19 [2]. There is numerous use of certain key points in the tweet text including punctuation marks, capitalization such as “GREAT”, intensifier such as “extremely”, emojis, conjunction such as “but”, and acronyms. VADER performs well with such key points in the tweet text. So, we choose VADER to extract the sentiment for our collected tweets over other salient sentiment analysis approaches. For sentiment analysis, we preprocess the collected tweets by removing only URLs and user mentions in the tweet text. Then we apply VADER on the preprocessed tweets to report sentiment scores. In particular, we report the VADER compound scores that are normalized to be between −1 (most extreme negative) and +1 (most extreme positive).

### D. COVID-19 KEYWORDS

Although we do not specify any keyword when collecting tweets, we needed to find tweets related to COVID-19 to compare them with the general background tweets. In this study, after analyzing sample tweets to find relevant keywords, we use the following 29 frequent keywords to extract COVID-19-related tweets (case-insensitive): covid, covid19, covid2019, covid_19, coronavirus, virus, COVD, COVD19, 2019nCoV, WuhanVirus, wuhan, SARSCoV2, SARS, novelcoronavirus, rona, therona, missrona, outbreak, pandemic, CoronavirusOutbreak, CoronavirusPandemic, CoronaOutbreak, SocialDistancing, StayAtHome, StayHome, quarantine, lockdown, mask, and WearAMask. When we search for COVID-19-related tweets, we exclude tweets related to the Corona beer. We label a tweet as COVID-19-related if its text contains any of the frequent keywords.

## IV. ANALYZING TWEETING PATTERNS

In this section, we describe tweeting patterns in the two states during the recognition period. We first show basic characteristics of the collected data such as the numbers of daily tweets and unique users in each state. We also compare word usage patterns in the two states and present county-level geospatial tweeting patterns. In addition, we describe tweeting patterns of COVID-19-related tweets only.

### A. GENERAL DATA PATTERNS

**FIGURE 2.**
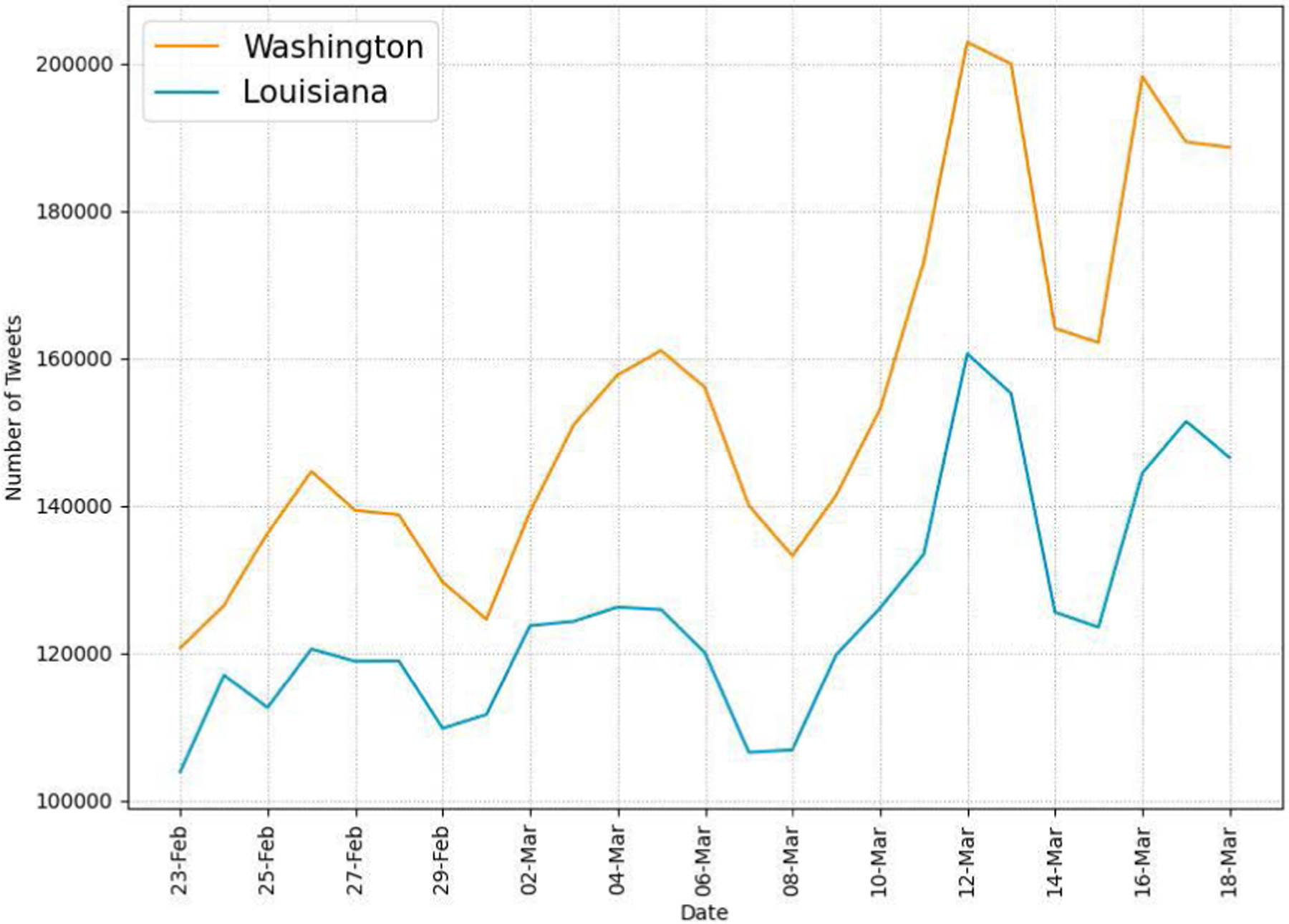
Daily numbers of tweets from WA and LA.

**FIGURE 3.**
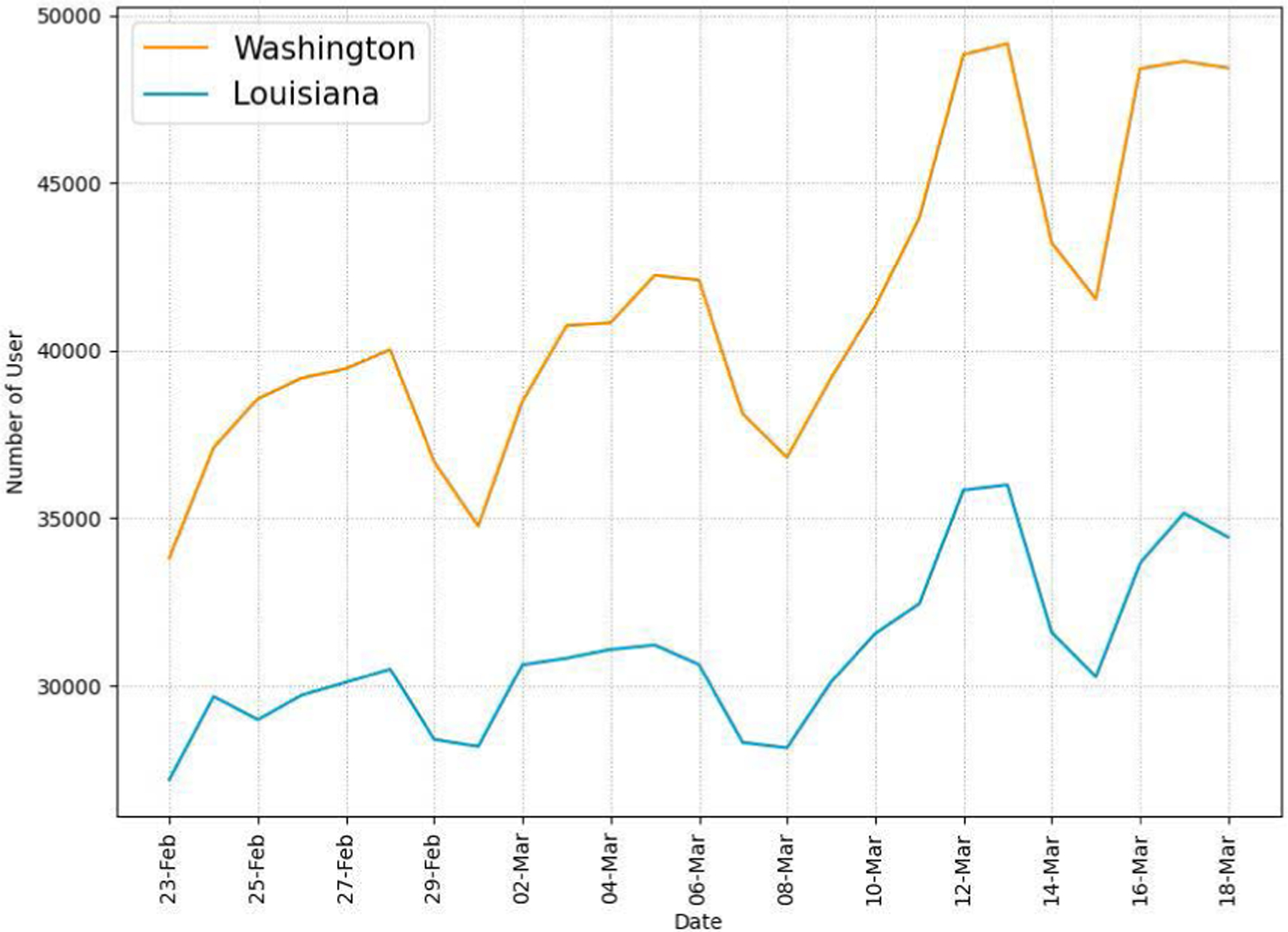
Daily numbers of unique active Twitter users from WA and LA.

We first report general data characteristics of collected Twitter data. We collect a total of 20,603,959 tweets during the recognition period using the data collection method described in Sec. III-B. For this study, we use language and geospatial operators to filter tweets from the collected Twitter data. Specifically, we use the lang: field to find English tweets only and the region: field in the derived user location to find tweets posted by users located in Louisiana or Washington. Through the filtering, we get a total of 7,004,155 tweets posted from the two states (3,870,660 from WA and 3,133,495 from LA). We use the sub_region: field in the derived user location to filter tweets posted from a specific county/parish. In addition to language and geospatial operators, to find COVID-19-related tweets, we use regular expression-based matching of 29 keywords in the tweet text. We find a total of 571,696 COVID-19-related tweets (378,517 and 193,179 tweets from WA and LA respectively). Fig. 2 shows the daily numbers of tweets in the two states during our study period. The figure presents increasing numbers of tweets in the two states as more users recognize the risk of COVID-19 during the recognition period. The drops on Feb. 29 - Mar. 1, Mar. 7–8, and Mar. 14–15 indicate less Twitter activity during the weekends. Fig. 3 shows the daily numbers of unique active Twitter users in the two states, indicating an increase in numbers of Twitter users during that same period.

**FIGURE 4.**
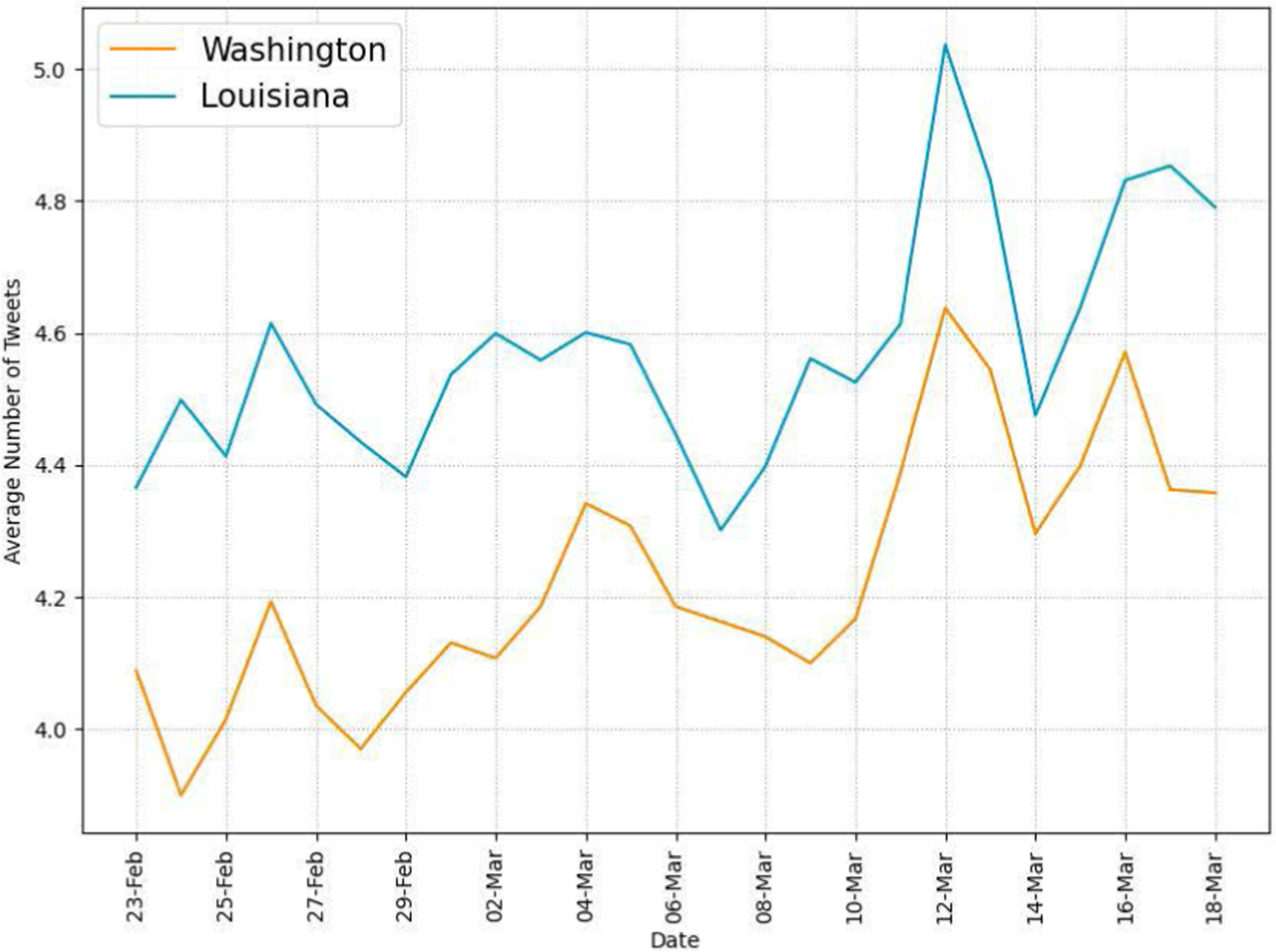
Average numbers of tweets per user in WA and LA.

Fig. 4 shows the daily average numbers of tweets per user in each state during the recognition period. The numbers show increased Twitter activity at the individual level as COVID-19 got more attention across both states. Table 2 presents the numbers of tweets, retweets, quote tweets, and replies in our collected data. About a half of the collected tweets are retweets, and the percentage of original tweets is about 10% in each state.

### B. WORD USAGE PATTERNS

**FIGURE 5.**
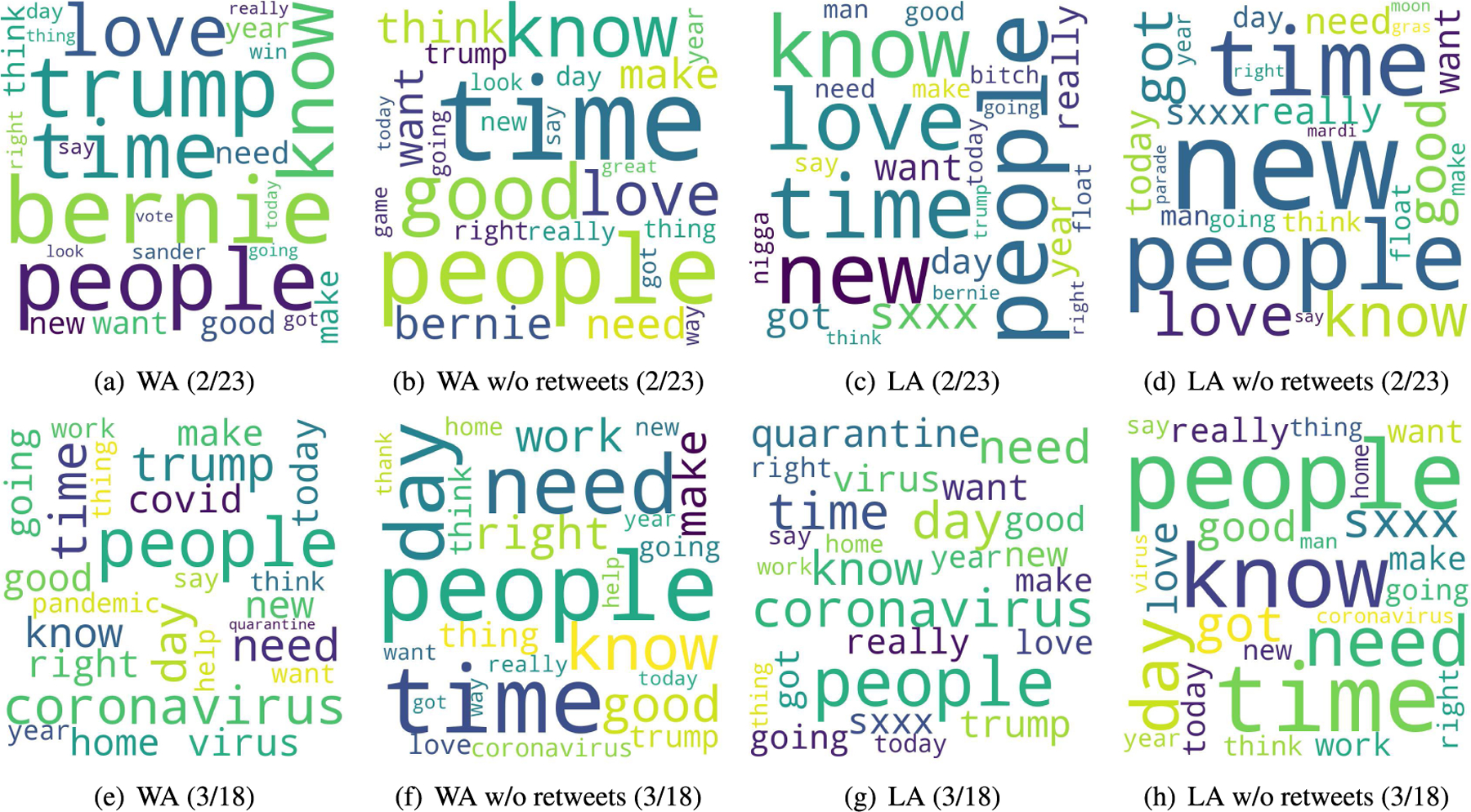
Word clouds.

Fig. 5 shows word clouds that visualize the most frequent words in our sample. We select the first (Feb. 23) and last (Mar. 18) days of our study period to highlight changes in the most frequent words during the recognition period. In addition to word clouds from all tweets, we generate word clouds by excluding retweets to analyze messages directly written by Twitter users in the two states. In addition to removing URLs and user mentions from the tweets, we preprocess tweets using a special word filter to generate word clouds. The special word filter removes collection words such as irrelevant short tweet terms (e.g., haha, yall), pronouns (e.g., myself, himself), articles (e.g., a, an, the), and other irrelevant stop-words that provide little information about content [18]. As shown in Fig. 5(e) and (g), Twitter users were frequently talking about COVID-19, such as “coronavirus” and “virus”, on Mar. 18 while there is no relevant frequent word for COVID-19 on Feb. 23 (Fig. 5(a) and (c)). It is interesting to note that there is no significant presence of COVID-19-related words in tweets directly written by users in the two states even on Mar. 18. For example, we can find “coronavirus” in a small font (i.e., less frequent word) in Fig. 5(f) and Fig. 5(h).

In addition to visualizing frequent words, we also use TFIDF (term frequency-inverse document frequency) scores to find important words in our tweet collection. TFIDF uses not only word frequency but also document frequency to give a lower score to words that appear in many tweets [19]. Before applying TFIDF, we preprocess the tweet text by removing emojis, stopwords, and collection words such as pronouns and adjectives. Tables 3 and 4 show the top-10 words based on TFIDF on Feb. 23 in Washington and Louisiana respectively. Similar to the word clouds, there is no word directly related to COVID-19 in the tables given that Feb. 23 is the first day of our recognition period. Tables 5 and 6 show the results for Mar. 18. From all tweets including retweets, we can find a couple of COVID-19-related words (i.e., coronavirus and quarantine) in the top-10 words. On the other hand, there are no COVID-19-related words in the results from tweets directly written by users (i.e., excluding retweets). We think that this is because Twitter users in the two states were consuming COVID-19 information posted by others rather than expressing their opinions during the recognition period.

### C. GEOSPATIAL PATTERNS

There are 39 counties in Washington and 64 parishes in Louisiana. According to the U.S. Census Bureau^5^ statistics for 2020, the total population is 7.705M and 4.658M in Washington and Louisiana respectively. We examine the geospatial characteristics of tweets from the two states during the recognition period. For county/parish-level comparisons, we analyze tweets that have county/parish information in the derived user location. In other words, we exclude tweets that have only state information for this geospatial analysis.

**FIGURE 6.**
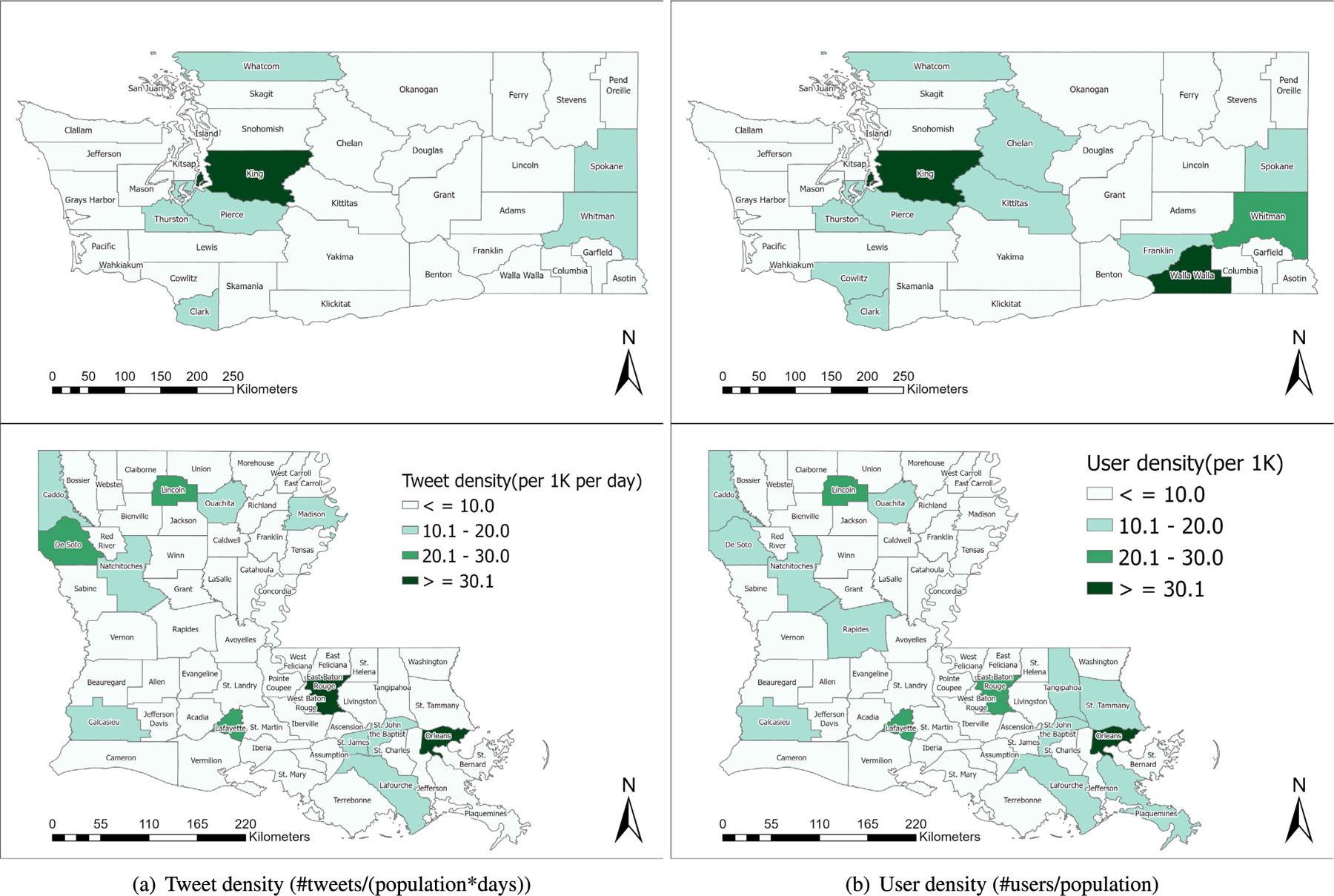
County-level tweet and user density in WA and LA.

Fig. 6(a) visualizes county-level tweet density (i.e., #tweets/(population*days)) in the two states during the entire recognition period. In Washington, the high tweet density in most of the urban and suburban counties indicates more Twitter activity than in rural counties. For example, King County, which includes Seattle, has the highest tweet density (2.3M tweets). It is worth noting that Whitman County, a rural county, shows a much higher tweet density (23K tweets) than other counties with a similar population. We think that this is because there is a large public university (Washington State University) in the county. A similar pattern of Twitter activities was found in Louisiana. For example, Orleans Parish including New Orleans has the highest tweet density (1.2M tweets). Fig. 6(b) visualizes county-level user density (i.e., #Twitter users/population), which shows similar geospatial patterns like tweet density for both states. It is interesting to note that, similar to Whitman County, Walla Walla County in WA where a few universities are located in shows high user density compared to other counties with a similar population.

We conduct a correlation study to understand relationships between county-level tweeting patterns such as tweet density, COVID-19 tweet density, and user density and socioeconomic factors such as median housing value, young population (age 15–29), median household income, and population density. Tables 7 and 8 show the Pearson correlation coefficient (r) between county-level socioeconomic factors and different tweeting variables in WA and LA respectively. Both tables demonstrate high positive correlations between tweeting variables and socioeconomic values in each state. Interestingly, median household income has no significant correlation with different tweeting variables in Louisiana.

### D. COVID-19-RELATED TWEETING PATTERNS

**FIGURE 7.**
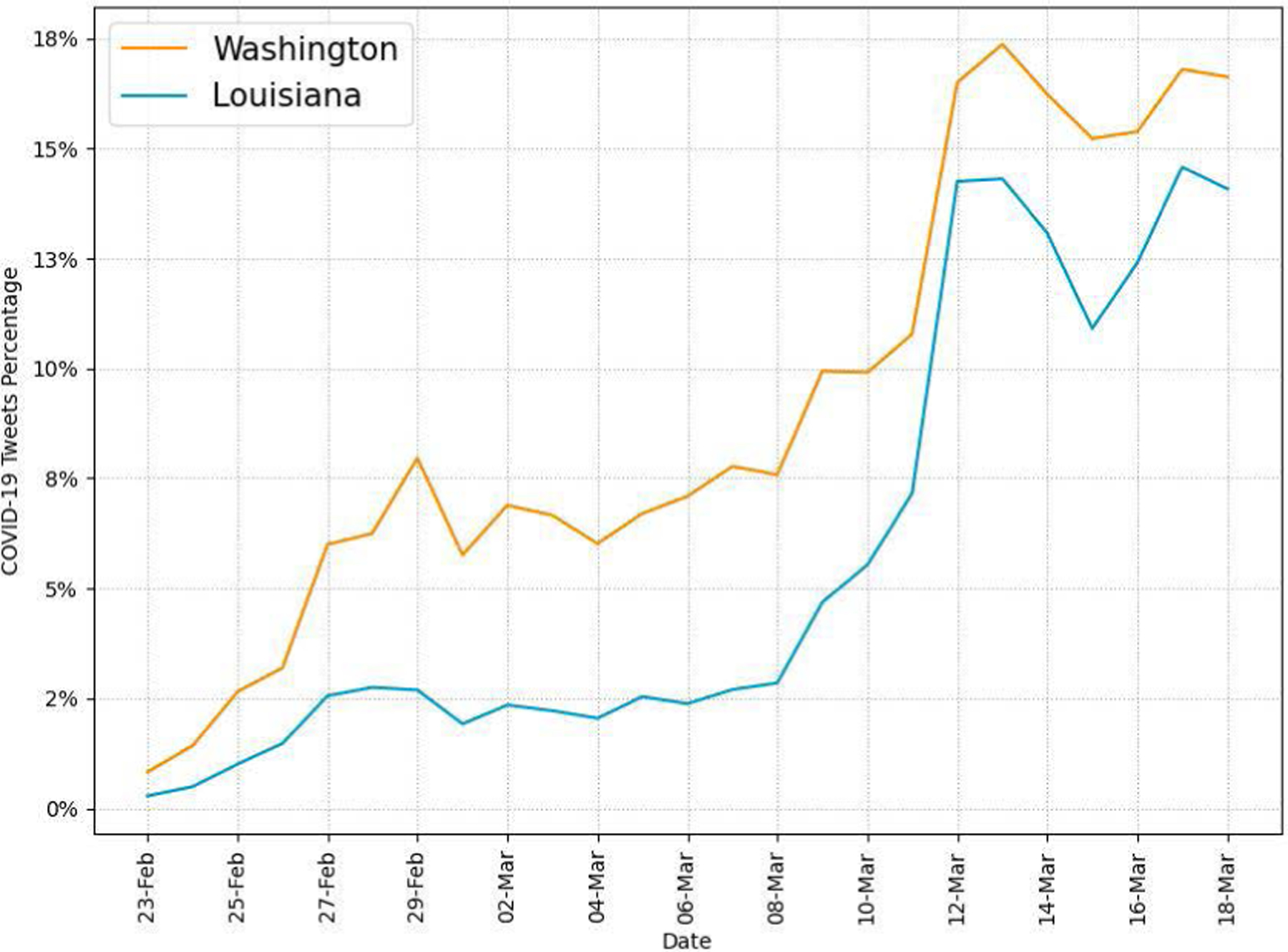
Daily COVID-19-related tweets.

Fig. 7 shows the daily percentages of tweets related to COVID-19 in WA and LA. We can find a surge in COVID-19-related tweets in both states from March 12 onward. From that date, the percentage of COVID-19-related tweets among all tweets is consistently higher than 10% (up to 16.3%) in both states. The significant increase of COVID-19-related tweets contributes to the overall increase in daily tweets shown in Fig. 2. The results show the rapid increase of awareness of COVID-19 from Twitter users in both states during the recognition period. The surge from March 12 is likely related to the pandemic declaration by WHO and the address by then U.S. President Donald J. Trump announcing travel restrictions from Europe and emergency action in the night on March 11.

**FIGURE 8.**
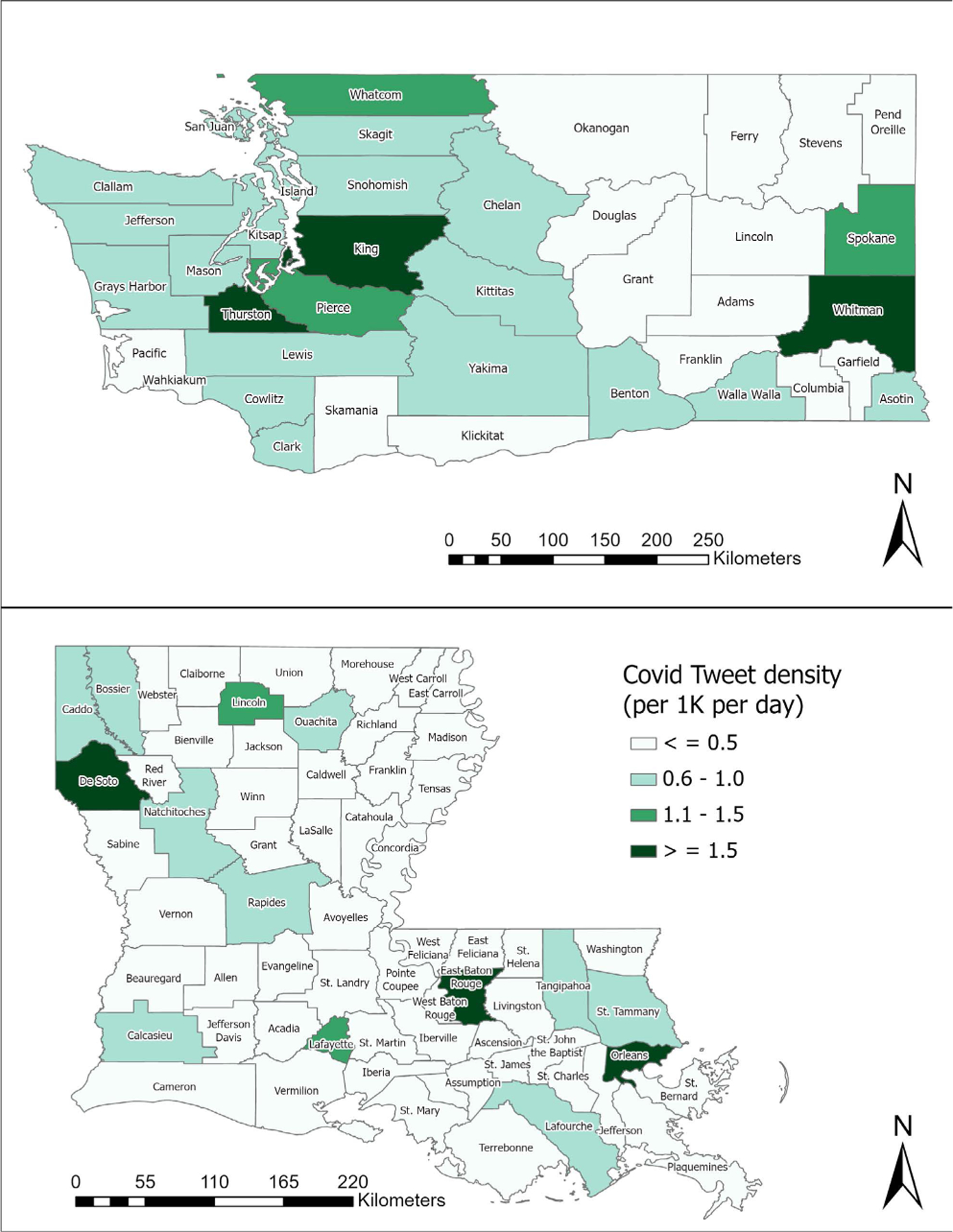
County-level COVID-19 tweet density (#COVID-19 tweets/(population*days)) in WA and LA.

Fig. 8 visualizes county-level COVID-19 tweet density (i.e., #COVID-19 tweets/(population*days)) in the two states during the entire recognition period. Like the tweet density, densely populated counties/parishes also show high COVID-19 tweet density.

## V. SENTIMENT ANALYSIS

In this section, we present inter- and intra-state sentiment variations during the recognition period. We also compare the sentiment of COVID-19-related tweets with that of all background tweets.

### A. STATE-LEVEL SENTIMENT ANALYSIS

**FIGURE 9.**
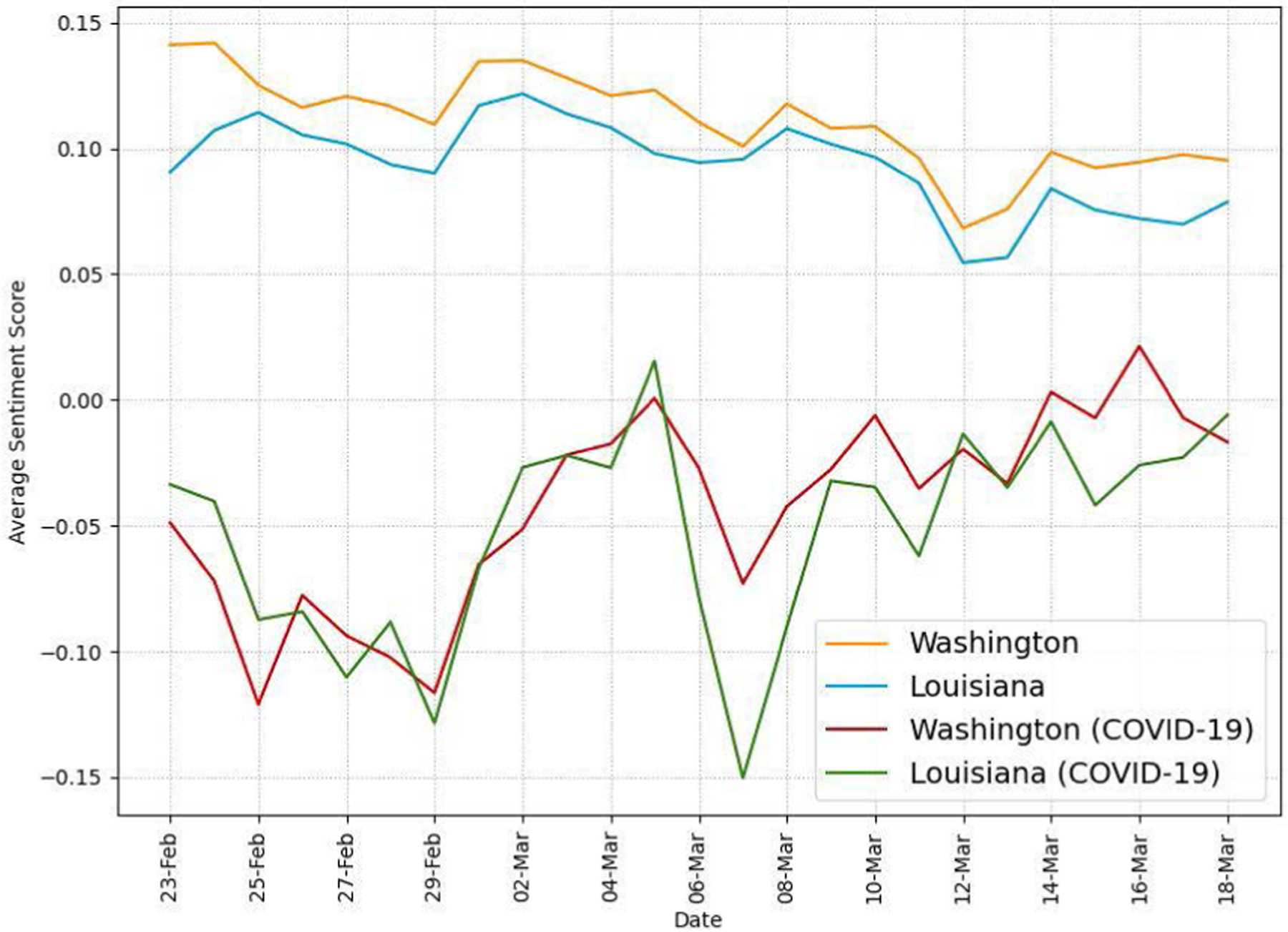
Daily average sentiment scores in WA and LA.

We compare the sentiment of our collected tweets between the two states. Fig. 9 shows the daily average sentiment scores, calculated using the VADER compound scores, in the two states during the recognition period. We include average sentiment scores for not only entire tweets but also only tweets related to COVID-19. The average sentiment scores for entire tweets are consistently in the positive range (i.e., higher than 0.05) while those for COVID-19-related tweets are in the neutral or negative range (i.e., lower than 0.05) in both states. We think that the relatively negative sentiment reflects growing public concerns about the pandemic. It is also interesting to note that the daily average sentiment of all tweets in LA is consistently lower than that in WA.

**FIGURE 10.**
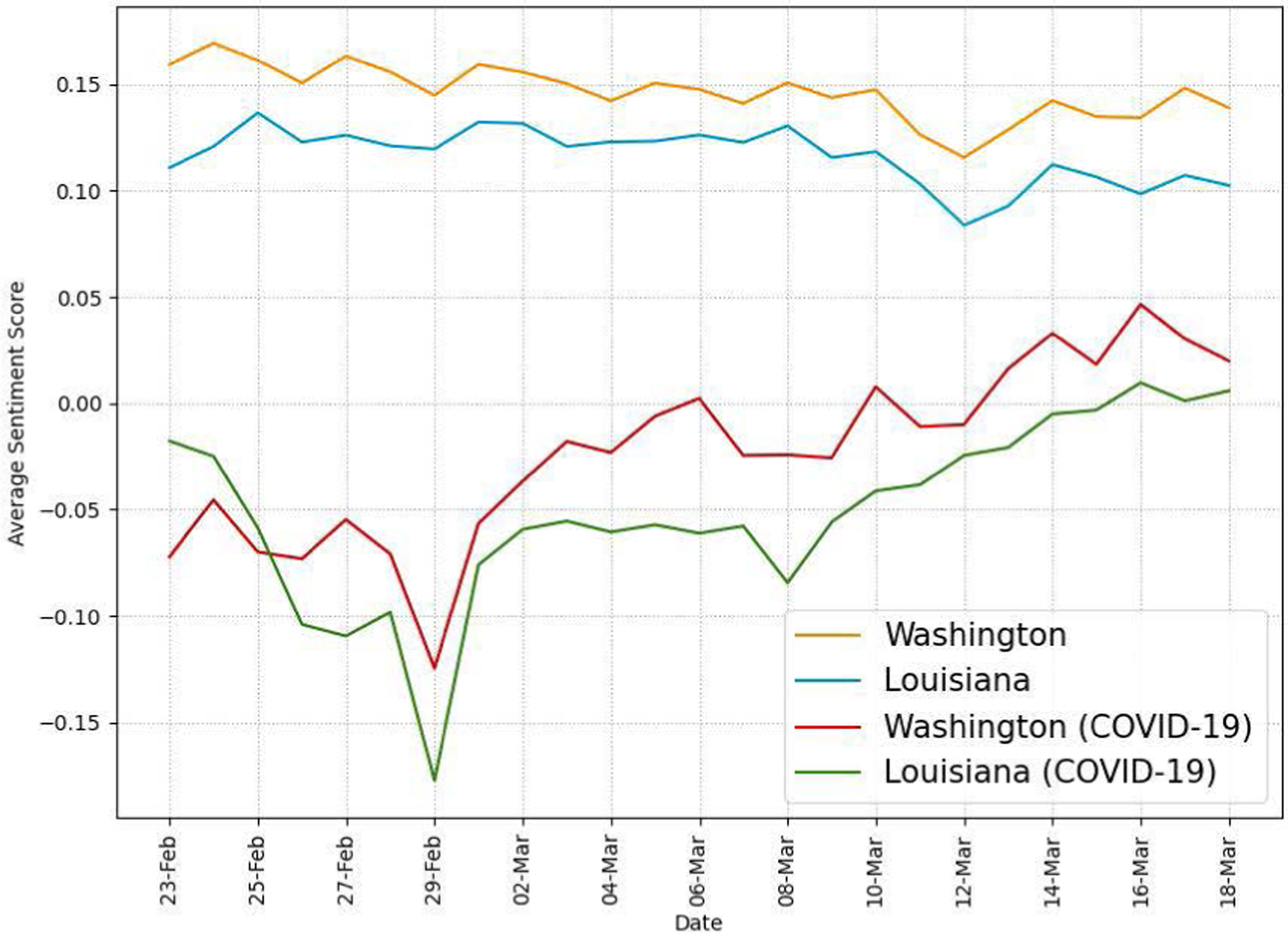
Daily average sentiment scores excluding retweets in WA and LA.

Fig. 10 shows the daily average sentiment scores of tweets excluding retweets to analyze messages directly written by the Twitter users in the two states. Like the results in Fig. 9, COVID-19-related tweets have consistently more negative sentiment scores than those of all tweets. It is interesting to note that both states have the most negative average sentiment scores on Feb. 29. We think that the first confirmed COVID-19 death in the U.S. on Feb. 29 contributed to the rise of negative sentiment on Twitter.

### B. COUNTY-LEVEL SENTIMENT ANALYSIS

**FIGURE 11.**
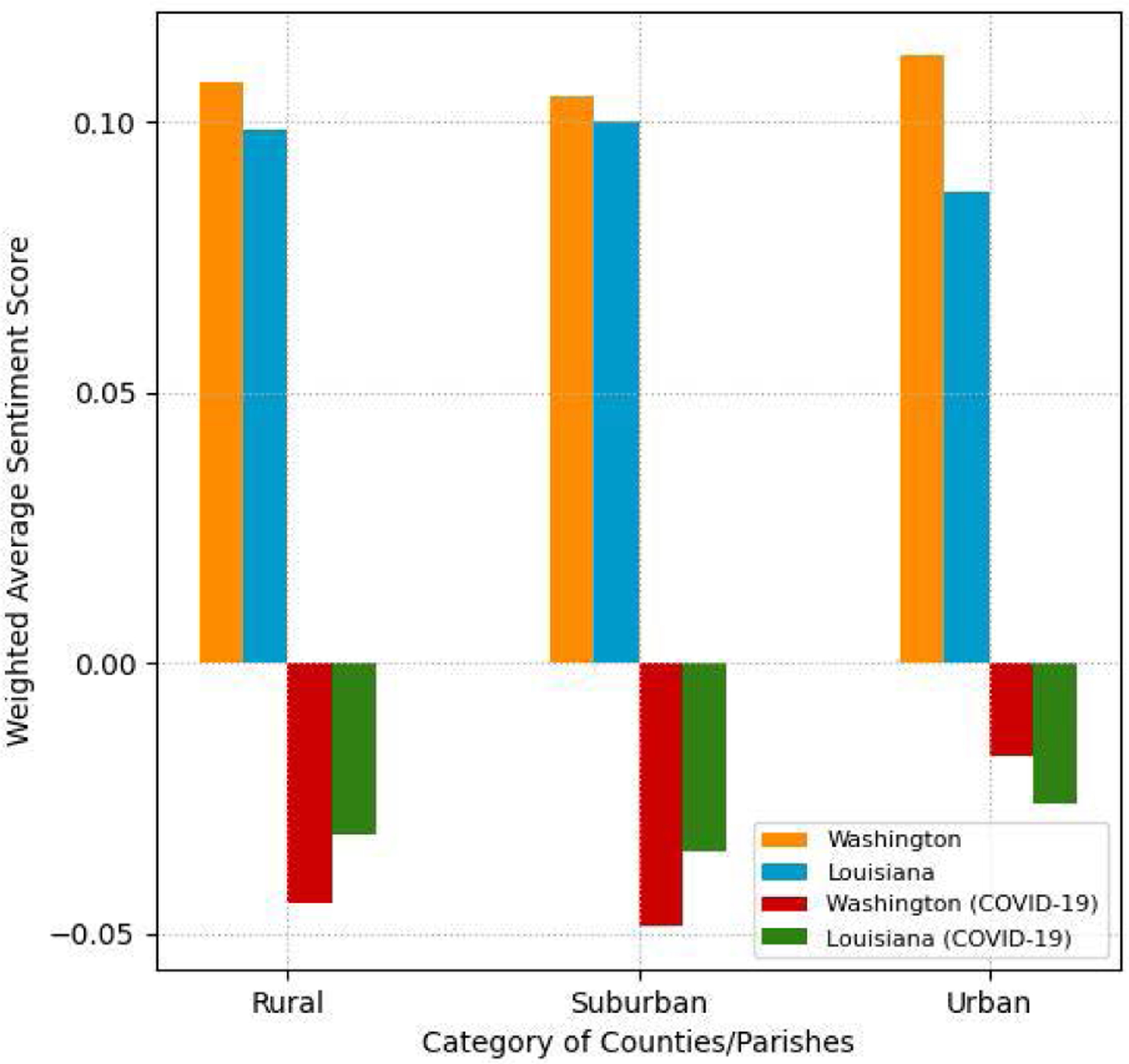
Weighted average sentiment scores of all tweets vs. COVID-19 tweets in rural, suburban, and urban counties or parishes.

To compare sentiment among counties/parishes, we categorize counties/parishes into three types based on their population density (i.e., population per sq. mile) as follows: urban (*density >* 500), suburban (100 *< density <*= 500), and rural (*density <*= 100). Fig. 11 shows the weighted average sentiment scores of all tweets and COVID-19-related tweets for the three categories in both states during the recognition period. For all tweets, the weighted average sentiment scores are positive in all categories of the two states. The result shows a higher positive weighted average sentiment score for urban counties in WA than in LA. On the other hand, for COVID-19-related tweets, the weighted average sentiment scores are negative for all categories in both states. The result also shows noticeably more negative weighted average sentiment scores for rural and suburban counties in WA than LA and the opposite pattern for urban counties.

In addition, we conduct a correlation study between county-level average sentiment scores and other tweeting and socioeconomic variables as shown in Table 9 and Table 10 for Washington and Louisiana respectively. The results demonstrate no significant correlation between sentiment scores and other variables in the two states. We think that this is because the recognition period was too early to capture meaningful county-wise disparities in terms of sentiment. Twitter users in the two states were likely consuming COVID-19 information rather than expressing their opinions during the recognition period, as evidenced in Fig. 5.

## VI. ANALYZING TWITTER ACTIVITIES RELATED TO GOVERNMENTAL ACCOUNTS

In this section, we analyze the Twitter activity of seven selected governmental accounts to understand their use of Twitter to disseminate public health information during the recognition period of the pandemic. For this analysis, we select Twitter handles of governors (GovInslee in WA and LouisianaGov in LA), public health departments (WADeptHealth in WA and LADeptHealth in LA), and emergency management divisions (EMD) in the two states (waEMD in WA and GOHSEP in LA) along with the Twitter handle of the federal-level Centers for Disease Control (CDC). Then we analyze public engagement related to the tweets posted by these accounts.

### A. TWEETING PATTERNS OF GOVERNMENTAL ACCOUNTS

**FIGURE 12.**
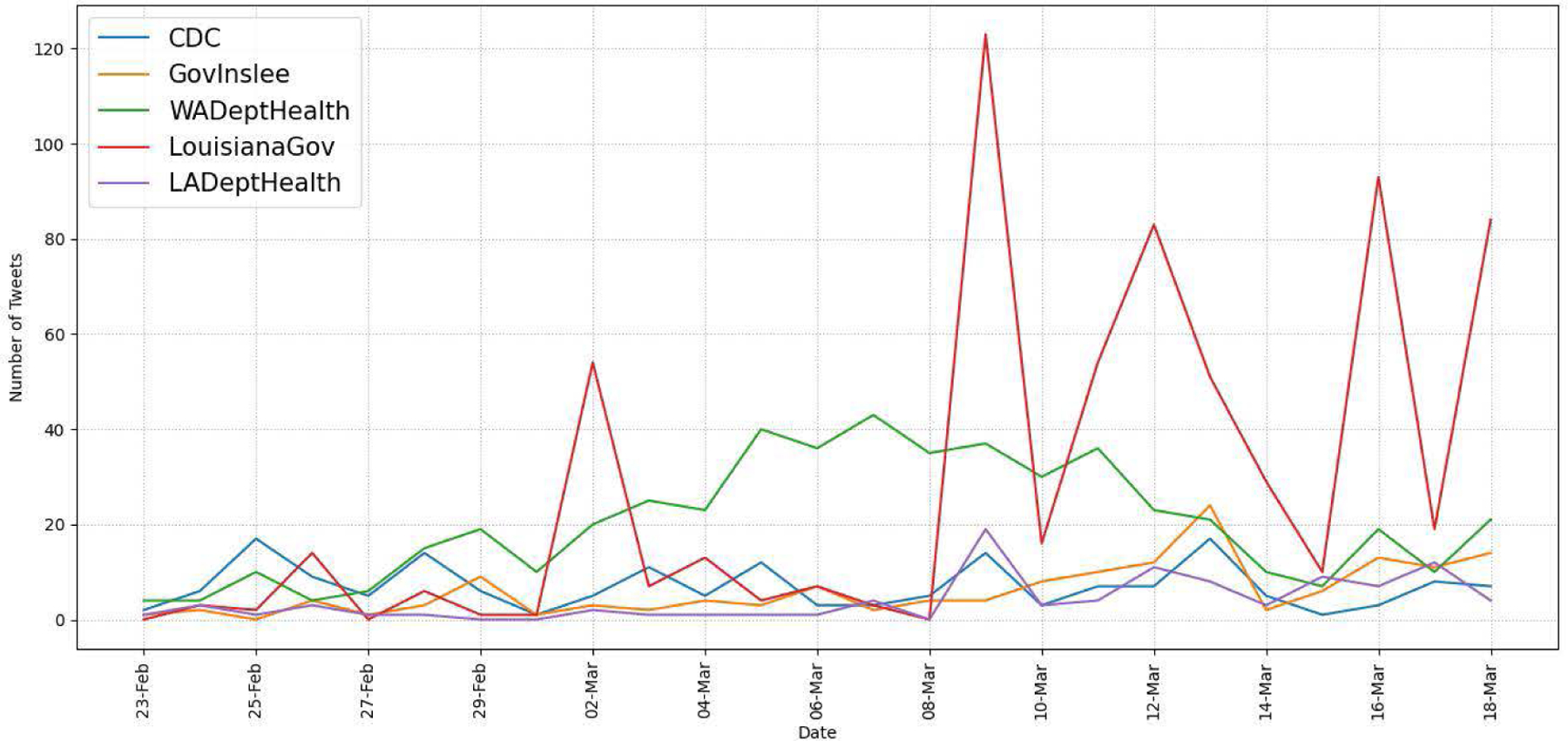
Daily tweet counts of governmental accounts.

**FIGURE 13.**
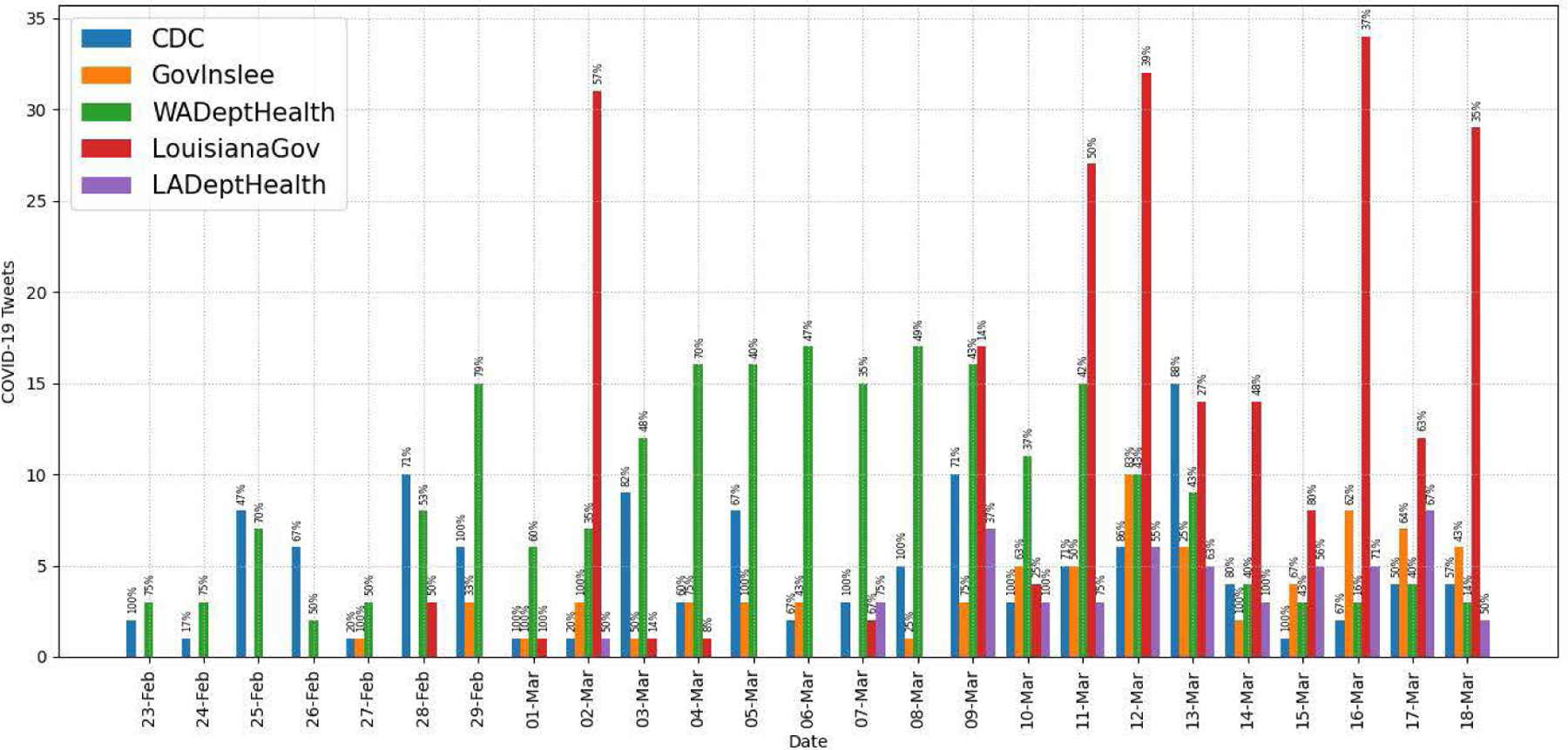
Daily COVID-19-related tweet counts of governmental accounts.

To collect entire tweets, including retweets, quote tweets, and replies posted by these seven accounts during the recognition period, we use the Academic Research Track API, which supports the full-archive search. Fig. 12 and Fig. 13 show the daily numbers of all tweets and COVID-19-related tweets respectively, posted by the governmental accounts. We exclude the numbers of the two EMD accounts in the figures for brevity. The results show that two Twitter handles, LouisianaGov and WADeptHealth, were more active than the others during the period. For further analysis, we also show the numbers of COVID-19-related tweets by tweet type (retweets, replies, or quote tweets) from the seven accounts in Table 11. Our analysis results indicate that they used Twitter differently for public health messaging during the recognition period of COVID-19. For example, although LouisianGov and waEMD posted more than 150 COVID-19-related tweets each during the period, most of them are retweets (75% and 69% respectively). WADeptHealth actively replied to messages posted by the users compared to the other accounts.

### B. PUBLIC ENGAGEMENT PATTERNS

We analyze the public engagement related to the COVID-19-related “original” tweets (i.e., excluding retweets, replies, and quote tweets) posted by the governmental Twitter accounts. Table 12 presents their average numbers of replies, quote tweets, retweets, and likes for COVID-19-related original tweets. It is interesting to note that the COVID-19-related original tweets from the WA governor (GovInslee) received much higher public engagement numbers than the other state-level accounts.

For further analysis, we extend our study on public engagement according to message function types to draw more insights. The literature on the use of organizational social media identified three primary message functions: information, action, and community [20]. We coded a total of 460 COVID-19-related tweets posted during the recognition period. We then selected 255 COVID-19-related “original” tweets, as shown in Table 12, for public engagement analysis. Tweet codes were adapted from the definitions of *information*, *action*, and *community* taken from [8] and [20]. Tweets were coded as *information* if they were one-way messages meant to provide information about COVID-19, including symptoms, risks, prevention, testing, and infection rates or case statistics. Tweets were coded as information if they focused on government actions or policies meant to address COVID-19 and if they referred readers to another governmental agency for COVID-19 information. We also included travel notifications as informational tweets, even if they included an imperative verb. In contrast, *action* tweets urged a particular behavior and mean to have readers enact the behavior in the tweet. These tweets use imperative verbs such as wash, stay, cover, employ, and avoid. They frequently include the word “should”—a sign a tweet is requesting action. *Community* tweets mean to compel readers to interact with each other or the tweeter. They often attempt to boost morale or use empathetic phrasing meant to provide emotional support. Following [20], these tweets do not necessarily expect a response but do attempt to strengthen the community. We coded any of the following tweet types as a community: tweets related to sympathy or death, Q&A tweet “events,” and tweets that encourage people to reach out to their neighbors or local community to make plans. Finally, some tweets contained text that could be coded as more than one of the three functions. For these tweets, following [8] we assigned a single code based on our assessment of the tweet’s primary purpose. To determine the primary purpose, we generally looked at what message was the majority of the text.

We found that the information function was the most commonly used message function by all the accounts. There are 198 (78%), 51 (20%), and six (2%) tweets related to information, action, and community function respectively. Table 13 shows the public engagement numbers by message function for the six state-level accounts and CDC. As expected in our hypothesis (H4), the action function has the highest engagement numbers in terms of the average retweet and like counts on all accounts except the health departments of both states (WADeptHealth and LADeptHealth).

### C. SENTIMENT ANALYSIS OF REPLIES TO GOVERNMENTAL ACCOUNTS

**FIGURE 14.**
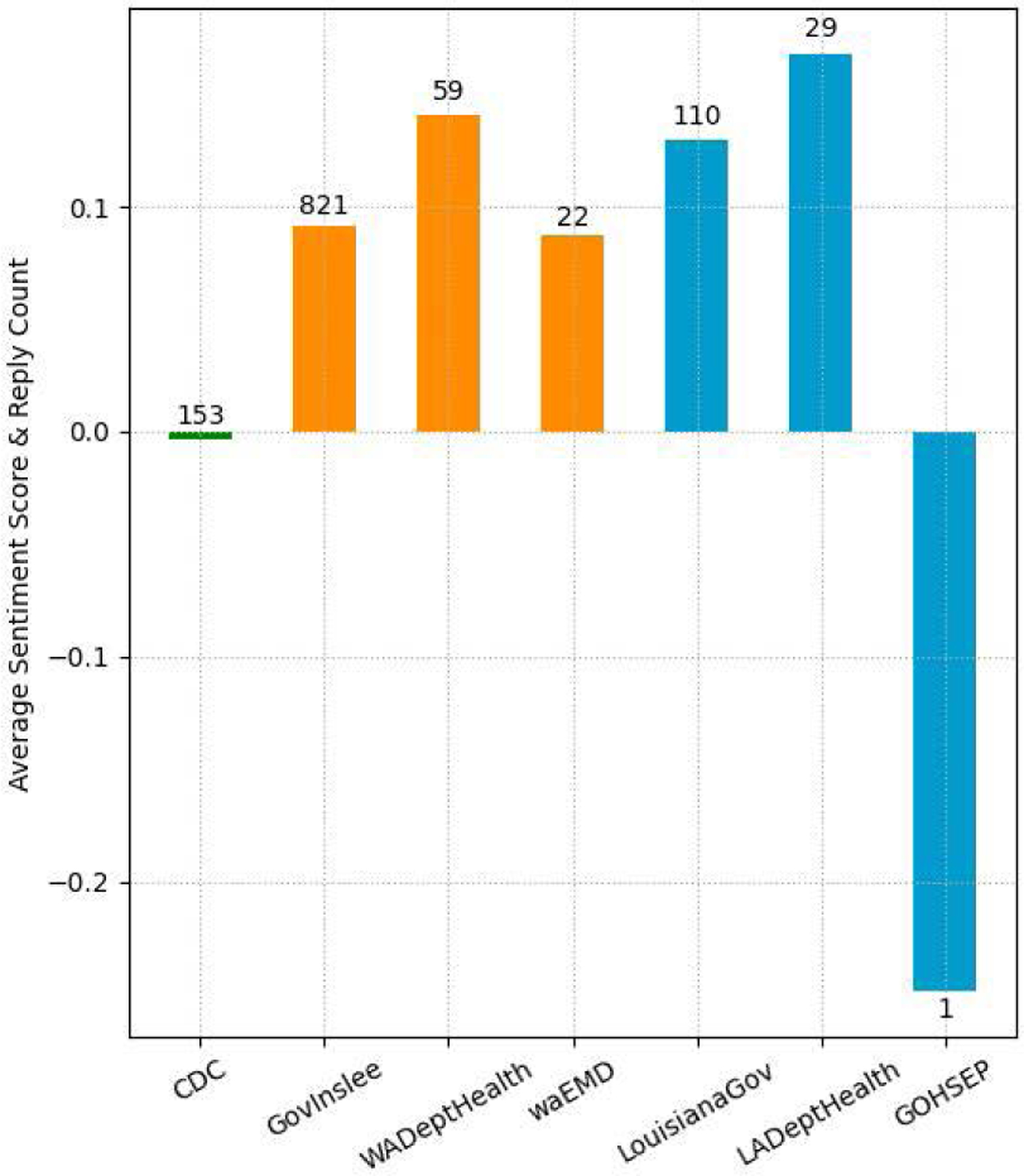
Average sentiment of replies to COVID related tweets by authorities.

We also analyze the sentiment of replies to tweets posted by the governmental Twitter accounts. For this analysis, we extract replies from our collected data (described in Sec. III-B). Fig. 14 presents the average sentiment scores of replies and reply counts found in our collected data to COVID-19-related tweets posted by the seven governmental accounts. Even though the results show that the replies to the health department have higher positive sentiments than those to the respective governor, there were not enough replies to make a definitive conclusion.

## VII. DISCUSSION

### A. RQ1: HOW DID TWITTER ACTIVITY, SENTIMENT, AND WORD USAGE VARY IN THE TWO STATES?

Our Twitter activity analysis results show that Washington had consistently more daily tweets and users than Louisiana during the recognition period although their variations displayed similar patterns, as shown in Figs. 2 and 3. The higher number of tweets and users in Washington was expected because it is a more populous state than Louisiana (7.7M vs. 4.7M as of 2020) and has the 15^*th*^ largest metropolitan area (Seattle) in the U.S. with an estimated population of 4M as of 2020. It is interesting to note that the average number of tweets per user was consistently higher in LA than in WA during the study period, as shown in Fig. 4. Our sentiment analysis results in Fig. 9 demonstrate that the average daily sentiment scores of all tweets in WA were consistently higher than those in LA during the recognition period although both states had sentiment scores in the positive range. When we consider only tweets directly posted by users (i.e., excluding retweets), the sentiment gap further increased, as evidenced in Fig. 10. In terms of word usage, the word clouds based on word frequency in Fig. 5 indicate similar frequent words in the two states although some political figure-related words, such as “Bernie” and “Trump”, were more frequently mentioned in WA than in LA.

### B. RQ2: TO WHAT EXTENT DID TWITTER ACTIVITY AND SENTIMENT VARY ACROSS COUNTIES OR PARISHES IN EACH STATE?

Previous research studies suggest that Twitter activity is positively correlated with population density [6]. Our correlation analysis results for counties/parishes also validate that Twitter activity (tweet density, COVID-19 tweet density, and user density) was positively correlated with population density in both states at the 0.01 level of significance, as shown in Tables 7 and 8. It is interesting to note that the correlation between Twitter activity and median household income was statistically insignificant in LA (Table 8) in contrast to significant positive correlations at the 0.01 level in WA (Table 7). In terms of county/parish-level sentiment variations, even though Fig. 11 shows less negative sentiment in urban counties/parishes, we could not find statistically significant correlations between average sentiment scores and socioeconomic conditions as shown in Tables 9 and 10.

### C. RQ3: HOW DID TWEETING PATTERNS DIFFER FOR COVID-19-RELATED TWEETS IN THE TWO STATES?

Our study reveals that there was more Twitter activity in Washington than in Louisiana in terms of the percentage of COVID-19-related tweets during the recognition period, as shown in Fig. 7. As we expected in hypothesis H1, we think that earlier exposure to COVID-19 cases in Washington led to more attention from Twitter users in the state. Our findings also show that the daily average sentiment scores of COVID-19-related tweets were in the negative range while those of all tweets were in the positive range in both states as shown in Fig. 9. It is interesting to note that, if we exclude retweets and focus only on tweets directly posted by users, the sentiment scores of tweets posted by users in Louisiana are consistently more negative (less positive) than those in Washington for both all and COVID-19-related tweets, as evidenced in Fig. 10. Our word usage analysis results based on word frequency and TFIDF scores suggest that there was no significant presence of COVID-19-related words in tweets directly written by users (i.e., those excluding retweets) in the two states even on the last day of our recognition period as shown in Fig. 5, Table 5, and Table 6. We think that this is because Twitter users in the two states were consuming COVID-19 information posted by others rather than expressing their opinions during the early period of the pandemic.

### D. RQ4: TO WHAT EXTENT DID GOVERNMENTAL TWITTER ACCOUNTS COMMUNICATE PUBLIC HEALTH MESSAGES AND DID THE USERS ON TWITTER ENGAGE WITH THE MESSAGES?

Our analysis results of seven governmental Twitter accounts indicate that they used Twitter differently for public health messaging during the recognition period of COVID-19. For example, as shown in Table 11, while the LA governor’s account (LouisianaGov) posted much more COVID-19-related tweets than the WA governor’s account (GovInslee), most (75%) of them were retweets. To analyze the public engagement related to tweets posted by the governmental accounts, we categorized their original COVID-19-related tweets by message function, and our analysis results show that the information function was the most commonly used message function (78% of all tweets evaluated). As we expected in hypothesis H4, governmental tweets with the action function had the highest engagement numbers in terms of the average retweet and like counts on all accounts but the account of both state health departments as shown in Table 13. Our findings suggest that they could post more COVID-19-related tweets with the action function to receive higher engagement from the users.

### E. RQ5: HOW DID SENTIMENT OF RESPONSES TO GOVERNMENTAL COVID-19-RELATED MESSAGES VARY ACROSS BOTH STATES?

Our sentiment analysis results of replies to COVID-19-related tweets posted by selected governmental accounts indicate that replies to the health department had higher positive sentiment than those to the respective governor, as shown in Fig. 14. However, we could not make a definitive conclusion because of the limited number of replies we could extract for certain governmental accounts. Our findings could be improved through additional reply collection during a longer period of time, and we leave this as our future work.

## VIII. CONCLUSION AND FUTURE WORK

In this study, we examined tweeting patterns and public engagement related to public health messaging in Washington and Louisiana during the 25-day recognition period of COVID-19. Our analysis results prove that Washington State had higher COVID-19-related tweet intensity by the users (H1), as evidenced in Fig. 7. However, tweeting patterns from selected governmental accounts, as shown in Fig. 13, do not present clear state-level differences because different accounts used different communication strategies regarding COVID-19. Our findings also validate that, at the county/parish level, the same pattern of Twitter use disparities between urban and rural found in previous studies existed in WA and LA (H2), as shown in Tables 7 and 8. In terms of county/parish-level sentiment disparities (H3), even though Fig. 11 shows less negative sentiment in urban counties/parishes, we could not find statistically significant correlations between average sentiment scores and socioeconomic conditions as shown in Tables 9 and 10. Our public engagement analysis results validate that governmental tweets with the action function have the highest engagement numbers in terms of average retweet and like counts on all accounts we evaluated except for each state’s health department Twitter account (H4). Although the governmental Twitter accounts we evaluated were active during the recognition period of the pandemic, our findings suggest that they could post more COVID-19-related tweets with the action function to receive higher engagement from users.

In our future work, we will extend our study to analyze both inter- and intra-surge disparities using more Twitter data covering wider periods. We will also apply large language models such as BERT to analyze the Twitter data and compare them with traditional approaches. In addition, we plan to study more focused topics related to COVID-19 on Twitter, such as misinformation/disinformation, media framing, and vaccine hesitancy.

## Figures and Tables

**TABLE 1.** COVID-19 timeline in WA and LA (1Q 2020).

Events	WA	LA
First confirmed case	Jan. 21	Mar. 9
First confirmed death	Feb. 29	Mar. 14
Confirmed cases exceeds 100	Mar. 7	Mar. 15
Confirmed cases exceeds 200	Mar. 10	Mar. 18
“Stay at home” order issued	Mar. 23	Mar. 22

**TABLE 2.** The numbers of tweets, retweets, quote tweets, and replies in WA and LA.

State	Population (2020)	#Tweets	#Retweets	#Quote Tweets	#Replies
Washington	7,705,281	3,870,660	1,893,385(49%)	507,400(13%)	1,106,244(29%)
Louisiana	4,657,757	3,133,495	1,701,201(54%)	478,760(15%)	571,866(18%)

**TABLE 3.** Top-10 words based on TFIDF on Feb. 23 (WA).

In All Tweets			In Without Retweets		
Word	TFIDF	Count	Word	TFIDF	Count
bernie	0.0121	5,972	love	0.0131	2,173
love	0.0116	3,577	good	0.0127	2,456
time	0.0112	4,469	time	0.0115	2,634
people	0.0108	5,084	know	0.0106	2,274
trump	0.0105	5,093	people	0.0097	2,578
know	0.0103	4,083	thank	0.0092	1,138
good	0.0100	3,515	think	0.0092	2,027
want	0.0092	3,490	want	0.0088	1,905
think	0.0086	3,318	need	0.0088	1,907
need	0.0084	3,283	right	0.0084	1,589

**TABLE 4.** Top-10 words based on TFIDF on Feb. 23 (LA).

In All Tweets			In Without Retweets		
Word	TFIDF	Count	Word	TFIDF	Count
love	0.0120	3,147	love	0.0135	1,608
sxxx	0.0111	3,103	know	0.0116	1,702
time	0.0106	3,388	time	0.0110	1,719
know	0.0105	3,166	got	0.0108	1,432
people	0.0104	3,789	good	0.0102	1,335
got	0.0103	2,937	new	0.0101	1,782
want	0.0102	2,844	people	0.0100	1,767
really	0.0092	2,659	sxxx	0.0096	1,207
good	0.0091	2,566	need	0.0095	1,247
need	0.0091	2,547	really	0.0094	1,264

**TABLE 5.** Top-10 words based on TFIDF on Mar. 18 (WA).

In All Tweets			In Without Retweets		
Word	TFIDF	Count	Word	TFIDF	Count
people	0.0137	10,926	time	0.0138	5,198
time	0.0135	9,664	people	0.0126	5,363
coronavirus	0.0133	10,137	good	0.0119	3,410
day	0.0131	7,984	day	0.0111	3,748
trump	0.0117	8,834	need	0.0111	3,827
need	0.0111	7,565	thank	0.0110	2,149
know	0.0101	6,304	know	0.0109	3,487
good	0.0095	5,206	love	0.0108	2,541
right	0.0087	5,208	right	0.0095	2,905
quarantine	0.0087	3,977	work	0.0083	2,861

**TABLE 6.** Top-10 words based on TFIDF on Mar. 18 (LA).

In All Tweets			In Without Retweets		
Word	TFIDF	Count	Word	TFIDF	Count
day	0.0145	5,905	time	0.0117	2,704
quarantine	0.0127	4,428	know	0.0114	2,275
people	0.0123	6,467	need	0.0113	2,230
time	0.0119	5,868	love	0.0109	1,676
know	0.0111	4,924	people	0.0109	2,727
coronavirus	0.0110	6,069	sxxx	0.0108	1,908
need	0.0110	4,842	good	0.0106	1,881
sxxx	0.0102	3,909	got	0.0106	1,936
love	0.0097	3,438	day	0.0100	2,046
want	0.0095	3,901	really	0.0089	1,659

**TABLE 7.** Pearson correlations between socioeconomic factors and tweeting variables (WA).

Tweeting Variables	Socioeconomic Factors			
	Median Housing Value	Young Population (age 15–29)	Median Household Income	Population Density
Tweet Density (per 1K)	0.636**	0.443**	0.591**	0.722**
COVID-19 Tweet Density (per 1K)	0.626**	0.421**	0.571**	0.713**
User Density (per 1K)	0.444**	0.631**	0.413**	0.515**

** Correlation is significant at the 0.01 level (2-tailed).

**TABLE 8.** Pearson correlations between socioeconomic factors and tweeting variables (LA).

Tweeting Variables	Socioeconomic Factors			
	Median Housing Value	Young Population (age 15–29)	Median Household Income	Population Density
Tweet Density (per 1K)	0.454**	0.301*	0.053	0.803**
COVID-19 Tweet Density (per 1K)	0.447**	0.234	0.054	0.820**
User Density (per 1K)	0.515**	0.386**	0.096	0.792**

** Correlation is significant at the 0.01 level (2-tailed).

*Correlation is significant at the 0.05 level (2-tailed).

**TABLE 9.** Pearson correlations between avg. sentiment scores and other variables in WA.

Variables	Sentiment	
	All Tweets	COVID-19 Tweets
Tweet Density(per 1K)	−0.152	0.160
COVID-19 Tweet Density (per 1K)	−0.184	0.143
User Density(per 1K)	−0.048	0.147
Median Housing Value	−0.0163	0.153
Young Population (age 15–29)	0.086	0.125
Median Household Income	−0.120	0.042
Population Density	−0.152	0.096

**TABLE 10.** Pearson correlations between avg. sentiment scores and other variables in LA.

Variables	Sentiment	
	All Tweets	COVID-19 Tweets
Tweet Density(per 1K)	−0.105	0.002
COVID-19 Tweet Density (per 1K)	−0.092	−0.020
User Density(per 1K)	−0.121	0.002
Median Housing Value	−0.022	−0.041
Young Population (age 15–29)	−0.072	0.052
Median Household Income	0.008	−0.115
Population Density	−0.097	0.009

**TABLE 11.** The numbers of COVID-19-related tweets by tweet type from governmental accounts.

Account	#Tweets	#Retweets	#Replies	#Quotes
CDC	120	6(5%)	25(21%)	4(3%)
GovInslee	75	5(7%)	15(20%)	11(15%)
WADeptHealth	225	100(44%)	73(32%)	12(5%)
waEMD	161	111(69%)	13(8%)	15(9%)
LouisianaGov	230	172(75%)	25(11%)	2(1%)
LADeptHealth	51	15(29%)	10(20%)	0(0%)
GOHSEP	50	43(86%)	0(0%)	0(0%)

**TABLE 12.** Public engagement statistics of COVID-19-related “original” tweets from governmental accounts.

Authority	Original Tweets	Avg. Retweets	Avg. Replies	Avg. Quotes	Avg. Likes
CDC	85	1,256.2	197.3	146.8	1,994.0
GovInslee	44	585.6	144.7	76.6	2,261.6
WADeptHealth	40	74.6	9.2	9.0	94.2
waEMD	22	51.6	10.4	6.1	93.6
LouisianaGov	31	260.5	40.8	64.3	594.9
LADeptHealth	26	68.0	10.3	10.6	68.2
GOHSEP	7	28.4	0.6	2.4	31.3

**TABLE 13.** Public engagement numbers by message function for seven governmental accounts.

Authority	Message Functions											
	Information				Action				Community			
	Avg. Retweets	Avg. Replies	Avg. Quotes	Avg. Likes	Avg. Retweets	Avg. Replies	Avg. Quotes	Avg. Likes	Avg. Retweets	Avg. Replies	Avg. Quotes	Avg. Likes
CDC	832.1	157.0	92.4	1,242.3	2,088.9	269.9	256.0	3,401.6	1,119.3	245	108	2,386.7
GovInslee	382.1	124.3	61.6	1,463.8	4,743	461	346	18,981	614	349	153	1,532
WADeptHealth	79.9	10.3	9.7	99.5	44.5	3	5.3	63.8	0	0	0	0
waEMD	45.2	11.9	6.1	91.9	68.8	6.2	6.3	98.2	0	0	0	0
LouisianaGov	127.9	15.9	33.7	169.1	775.8	141	182.7	2,318.7	239.5	27.5	60.5	320
LADeptHealth	70.5	10.7	11.0	70.9	5	1	0	1	0	0	0	0
GOHSEP	26.4	0.8	1.2	29	33.5	0	5.5	37	0	0	0	0

## Footnotes

1
https://thehill.com/policy/technology/508670-twitter-usage-growth-surges-amid-coronavirus-pandemic

2
https://developer.twitter.com/en/solutions/academic-research

3
https://developer.twitter.com/en/docs/tutorials/filtering-tweets-by-location

4
https://developer.twitter.com/en/docs/twitter-api/enterprise/enrichments/overview/profile-geo

5
https://www.census.gov/library/stories/state-by-state/
